# The impact of cannabis legalization for recreational purposes: The Canadian experience

**DOI:** 10.1192/j.eurpsy.2023.81

**Published:** 2023-07-19

**Authors:** B. Le Foll

**Affiliations:** ^1^ Translational Addiction Research Laboratory, Centre for Addiction and Mental Health; ^2^Department of Psychiatry, Faculty of Medicine; ^3^Department of Pharmacology and Toxicology; ^4^Departments of Family and Community Medicine; ^5^Institute of Medical Science, University of Toronto, Toronto; ^6^Waypoint Research Institute, Waypoint Centre for Mental Health Care, Penetanguishene, Canada

## Abstract

**Abstract:**

Cannabis Legalization for Recreational Purposes took place in Canada in October 2018. One of the federal government’s stated goals with this legalization was to protect Canadian youth from cannabis-related harms. The Canadian model differs from other jurisdictions that legalized recreational cannabis use, especially with regard to a higher degree of government regulation of the cannabis market. Another difference is the development and endorsement of lower-risk cannabis use guidelines to educate the public and health professionals. Here, we will present the changes in the regulation of the Canadian cannabis market. We will also present some changes in the epidemiology and parameters of cannabis use (modes of use, potency of cannabis) among adults and youths. Although it is clear that prevalence of use has increased in some groups (notably older adults), results for youth are mixed, with the majority of studies showing no pronounced increase. A trend of a decrease in youth cannabis use seen pre-legalization may have reversed. Data about changes in the age of initiation, the influence of legalization on sex and gender, and race/ ethnicity are limited, with evidence suggesting that the age of initiation slightly increased and the prevalence of use has become more similar between females and males. The development and utility of the lower-risk cannabis use guidelines will be also presented.

**Disclosure of Interest:**

B. Le Foll Grant / Research support from: . Dr. Le Foll has in-kind donations of cannabis products from Aurora Cannabis Enterprises Inc. . Dr. Le Foll has obtained industry funding from Canopy Growth Corporation (through research grants handled by the Centre for Addiction and Mental Health and the University of Toronto)

